# Redox Stress Biomarkers and Their Association With Renal Dysfunction in Chronic Kidney Disease

**DOI:** 10.7759/cureus.108120

**Published:** 2026-05-01

**Authors:** Sri Lakshmi Kothakapa, Sulekha Ramireddy, Sanjay Reddy Thanugundla, Parimi Vamsi Krishna, Sainithya Chittireddy, Muralidhar Chinnapaka

**Affiliations:** 1 Internal Medicine, Malla Reddy Institute of Medical Sciences, Hyderabad, IND; 2 General Medicine, Malla Reddy Institute of Medical Sciences, Hyderabad, IND; 3 Internal Medicine, J.J.M. Medical College, Davangere, IND; 4 Pharmacology, Government Medical College and Hospital, Maheshwaram, Hyderabad, IND

**Keywords:** antioxidants, catalase, chronic kidney disease, malondialdehyde, nitric oxide, nitrosative stress, oxidative stress, sod

## Abstract

Background: Chronic kidney disease (CKD) is characterised by progressive loss of renal function and is closely associated with enhanced oxidative and nitrosative stress. Excess production of reactive oxygen and nitrogen species contributes to cellular injury, inflammation, and accelerated disease progression. This study aimed to evaluate oxidative and nitrosative stress markers in CKD patients and compare them with healthy controls.

Methods: A hospital-based case-control study was conducted, including 80 diagnosed CKD patients and 80 age- and sex-matched healthy individuals. Blood samples were analysed for markers of oxidative stress, such as malondialdehyde (MDA), superoxide dismutase (SOD), and catalase, along with nitrosative stress marker nitric oxide (NO). Standard biochemical methods were used for estimation. Statistical analysis was performed using appropriate parametric and non-parametric tests, with p < 0.05 considered significant.

Results: Patients with CKD demonstrated significantly greater oxidative and nitrosative stress than healthy controls, as reflected by higher malondialdehyde levels (5.8 ± 1.2 vs. 2.9 ± 0.8 nmol/mL, p < 0.001) and nitric oxide levels (62.4 ± 10.5 vs. 34.7 ± 7.9 µmol/L, p < 0.001). Antioxidant enzyme activity was significantly reduced in the CKD group, with superoxide dismutase measuring 1.9 ± 0.6 U/mL compared with 3.8 ± 0.9 U/mL in controls, and catalase measuring 28.5 ± 6.7 kU/L compared with 46.2 ± 8.1 kU/L, respectively (p < 0.001 for both). Estimated glomerular filtration rate was negatively correlated with malondialdehyde levels among CKD patients, suggesting that oxidative stress increased as renal function declined (r = −0.62, p < 0.001).

Conclusion: The findings indicate a pronounced imbalance between oxidant production and antioxidant defence in CKD. Elevated oxidative and nitrosative stress may play a key role in disease progression and complications. Monitoring these biomarkers could aid in early intervention and therapeutic strategies targeting oxidative damage.

## Introduction

Chronic kidney disease (CKD) is a major global health concern, characterised by a gradual and irreversible decline in renal function, leading to significant morbidity and mortality. It is increasingly recognised as a systemic disorder that affects multiple organ systems, particularly the cardiovascular system, thereby contributing to a high burden of complications and reduced quality of life [1,2]. The progression of CKD is influenced by several interrelated mechanisms, among which oxidative and nitrosative stress have emerged as central contributors to renal injury and disease advancement [3].

Oxidative stress refers to a state in which the generation of reactive oxygen species (ROS) exceeds the capacity of endogenous antioxidant defence systems. Under physiological conditions, ROS plays a role in cellular signalling and homeostasis; however, excessive production leads to damage of lipids, proteins, and nucleic acids, ultimately impairing cellular function [4,5]. In CKD, multiple factors, including uremic toxin accumulation, mitochondrial dysfunction, chronic inflammation, and activation of pro-oxidant enzymes such as NADPH oxidase, contribute to enhanced ROS production [6,7]. This imbalance is further aggravated by a decline in antioxidant mechanisms, creating a sustained pro-oxidant environment.

In addition to oxidative stress, nitrosative stress also appears to have an important role in the pathophysiology of CKD. This form of cellular injury is mainly driven by reactive nitrogen species (RNS), especially nitric oxide (NO) and its reactive derivatives. Under normal physiological conditions, NO helps maintain vascular tone, supports renal blood flow, and protects endothelial function. However, in CKD, its metabolism becomes disturbed due to persistent inflammation, oxidative burden, and impaired endothelial regulation. One important mechanism is endothelial nitric oxide synthase (eNOS) uncoupling, where the enzyme produces superoxide instead of biologically useful NO. This reduces NO bioavailability and simultaneously increases free radical generation. Superoxide can further react with NO to form peroxynitrite, a highly reactive molecule capable of damaging lipids, proteins, DNA, and mitochondrial components. These changes promote endothelial dysfunction, vascular stiffness, inflammatory activation, and microvascular injury, all of which may contribute to progressive renal damage and cardiovascular complications commonly seen in CKD patients [8,9]. The combined effect of ROS and RNS leads to disruption of redox signalling pathways, promoting apoptosis, fibrosis, and progressive nephron loss.

Emerging evidence suggests that oxidative and nitrosative stress are closely linked with chronic inflammation, forming a vicious cycle that accelerates CKD progression. Persistent inflammatory stimuli increase cytokine production and oxidative burden, while oxidative stress further amplifies inflammatory signalling pathways such as NF-κB and TGF-β, contributing to renal fibrosis [3,10]. Moreover, mitochondrial dysfunction and impaired mitophagy have been identified as key mechanisms that perpetuate oxidative damage in CKD, leading to further deterioration of renal function [11].

The clinical significance of oxidative stress in CKD extends beyond renal injury, as it is strongly associated with cardiovascular complications, endothelial dysfunction, and increased mortality. Biomarkers such as malondialdehyde, advanced oxidation protein products, and nitric oxide metabolites have been shown to correlate with disease severity and progression [12,13]. Despite growing understanding, therapeutic strategies targeting oxidative stress have yielded variable outcomes, highlighting the need for further investigation into the underlying mechanisms and potential interventions [14-19].

Based on this background, the present study was planned with clear objectives. The primary objective was to evaluate oxidative and nitrosative stress status in patients with CKD by estimating malondialdehyde and nitric oxide levels. The study also aimed to assess antioxidant defence by measuring superoxide dismutase and catalase activity. In addition, these biomarkers were compared between CKD patients and healthy controls, and their relationship with renal function parameters, particularly serum creatinine, blood urea, and estimated glomerular filtration rate (eGFR), was examined. Through these objectives, the study sought to understand how redox imbalance is associated with the severity of renal dysfunction and whether these markers may have potential relevance in assessing CKD progression.

## Materials and methods

Study design and setting

This investigation was designed as a hospital-based case-control study conducted at Malla Reddy Institute of Medical Sciences, Hyderabad, a tertiary-care teaching hospital serving a diverse patient population. The study was conducted over one year, from January 2025 to December 2025. The primary objective was to evaluate oxidative and nitrosative stress in patients with CKD and to compare these parameters with those in healthy individuals.

Study population

A total of 160 participants were included in the study and were categorised into two groups. The case group comprised 80 patients diagnosed with CKD, recruited from the nephrology outpatient department and inpatient wards. The control group consisted of 80 apparently healthy individuals drawn from hospital staff and patient attendants, matched for age and gender to minimise bias. All participants were enrolled after careful screening and fulfilment of eligibility criteria.

Inclusion criteria

Participants aged between 18 and 70 years with a confirmed diagnosis of CKD were included. CKD was defined based on reduced kidney function, indicated by an estimated glomerular filtration rate (eGFR) of less than 60 mL/min/1.73 m² persisting for more than three months. Only those who provided informed consent and were clinically stable at the time of sampling were considered for inclusion.

Exclusion criteria

Patients with acute kidney injury, recent infections, chronic inflammatory or autoimmune disorders, malignancy, and known liver disease were excluded. Individuals receiving antioxidant therapy, vitamin supplements, or drugs known to influence oxidative status were also excluded. Additional exclusions included smokers, chronic alcohol users, and those with recent surgical interventions, as these factors could independently alter oxidative and nitrosative stress markers.

Ethical considerations

The study protocol was reviewed and approved by the Institutional Ethics Committee of Malla Reddy Institute of Medical Sciences. All procedures were conducted in accordance with ethical standards for human research. Written informed consent was obtained from each participant after explaining the purpose and nature of the study in a language they understood. Participant confidentiality was ensured by anonymising data and restricting access to authorised personnel only.

Sample size calculation

The required sample size was determined for a two-group comparison of continuous biochemical parameters. Using a two-tailed hypothesis, an alpha level of 0.05, a power of 80%, and an anticipated moderate effect size (Cohen’s d = 0.5), the estimated minimum sample size was 64 subjects per group. After allowing for potential dropouts, exclusions, and incomplete laboratory data, the final sample size was increased to 80 participants in each group. Thus, the study included 80 CKD patients and 80 healthy controls.

Clinical evaluation and data collection

A detailed clinical history was obtained from all participants, including duration of CKD, associated comorbidities such as diabetes mellitus and hypertension, and medication history. Physical examination was performed, and relevant anthropometric measurements were recorded. Clinical staging of CKD was done based on eGFR values.

Sample collection and processing

After an overnight fast of 8-10 hours, approximately 5 mL of venous blood was collected under aseptic precautions. The sample was divided into plain and anticoagulant-containing tubes. Serum was separated by centrifugation at 3000 rpm for 10 minutes. The aliquots were stored at −20°C and analysed within a specified time period to prevent degradation of oxidative stress markers. All pre-analytical variables were carefully controlled to ensure consistency.

Biochemical analysis

Routine renal function parameters were estimated from serum samples using an automated clinical chemistry analyser. Blood urea was measured by the urease-glutamate dehydrogenase method, and serum creatinine was estimated by the modified Jaffe kinetic method or enzymatic method, according to the standard laboratory protocol. Commercially available reagent kits from Erba Mannheim, Transasia Bio-Medicals Ltd., India, were used for routine biochemical analysis. The estimated glomerular filtration rate was calculated using the CKD-EPI equation, which is widely used for the assessment of renal function in adult patients [20].

Estimation of oxidative stress markers

Lipid peroxidation was assessed by measuring serum malondialdehyde using the thiobarbituric acid reactive substances method [21]. The assay was performed using the TBARS Assay Kit (Cayman Chemical Company, Ann Arbor, MI, USA; Catalogue No. 10009055). In this method, malondialdehyde reacts with thiobarbituric acid under acidic, high-temperature conditions to form a pink-coloured MDA-TBA complex. The absorbance was measured spectrophotometrically, and values were expressed as nmol/mL.

Superoxide dismutase activity was estimated using the Superoxide Dismutase Assay Kit (Cayman Chemical Company, Ann Arbor, MI, USA; Catalogue No. 706002). The assay is based on the ability of SOD to inhibit superoxide-mediated reactions, and enzyme activity was expressed as units per millilitre. Catalase activity was measured using the Catalase Assay Kit (Cayman Chemical Company, Ann Arbor, MI, USA; Catalogue No. 707002). This assay measures the decomposition of hydrogen peroxide, and catalase activity was expressed as kilounits per litre. Superoxide dismutase and catalase were selected because they are important endogenous antioxidant enzymes involved in protection against free radical-mediated cellular injury.

Estimation of nitrosative stress marker

Nitrosative stress was assessed by estimating serum nitric oxide indirectly through its stable metabolites, mainly nitrite and nitrate. The estimation was carried out using the Nitrate/Nitrite Colorimetric Assay Kit (Cayman Chemical Company, Ann Arbor, MI, USA; Catalogue No. 780001), based on the Griess reaction [22]. In this method, nitrite reacts with Griess reagent to form a coloured azo compound, which is measured spectrophotometrically. The results were expressed as µmol/L. Since nitric oxide is unstable in biological fluids, estimation of its stable end products provides a practical measure of systemic nitric oxide production.

Quality assurance and control

All biochemical estimations were carried out under standard laboratory conditions using calibrated instruments, validated procedures, and commercially available reagents or assay kits. The manufacturer, catalogue number, batch number, and expiry date of each reagent or kit were recorded before analysis. Routine renal function tests were performed using reagent kits from Erba Mannheim (Transasia Bio-Medicals Ltd., India). Oxidative and nitrosative stress markers were estimated using assay kits from Cayman Chemical Company, Ann Arbor, MI, USA, including the TBARS Assay Kit (Catalogue No. 10009055), Superoxide Dismutase Assay Kit (Catalogue No. 706002), Catalase Assay Kit (Catalogue No. 707002), and Nitrate/Nitrite Colorimetric Assay Kit (Catalogue No. 780001).

Internal quality control samples were run with each analytical batch to ensure accuracy and reproducibility. Instrument calibration was performed according to the manufacturer’s recommendations. Selected samples were analysed in duplicate to assess precision. Repeat analysis was performed when duplicate readings showed unacceptable variation or when values were outside the expected analytical range. Intra-assay and inter-assay coefficients of variation were monitored using quality control material and were maintained within acceptable laboratory limits.

Statistical analysis

Data were entered into Microsoft Excel (Microsoft Corp., Redmond, WA) and analysed using SPSS software, version 23.0 (IBM Corp., Armonk, NY). Continuous variables were expressed as mean ± standard deviation, while categorical variables were presented as frequencies and percentages. The distribution of continuous variables was assessed before selecting the appropriate statistical test. Normally distributed variables were compared between CKD patients and healthy controls using the independent sample t-test. For non-normally distributed variables, the Mann-Whitney U test was applied.

Categorical variables were compared using the chi-square test or Fisher’s exact test, as appropriate. Pearson’s correlation analysis was used to assess the association between oxidative and nitrosative stress markers and renal function parameters such as serum creatinine, blood urea, and estimated glomerular filtration rate. Spearman’s correlation was considered for non-parametric data. A p-value of less than 0.05 was considered statistically significant. The strength and direction of correlation were interpreted using the correlation coefficient along with the corresponding p-value.

## Results

Baseline characteristics of study participants

The study included 160 participants, with 80 CKD patients and 80 healthy controls. The mean age of CKD patients was 52.6 ± 11.4 years, which was comparable to controls (50.8 ± 10.9 years; p = 0.312). The gender distribution was similar in both groups (male:female ratio = 1.3:1 in CKD vs 1.2:1 in controls; p = 0.842). A higher proportion of CKD patients had comorbid conditions such as hypertension (62.5%) and diabetes mellitus (48.8%) (Table 1).

**Table 1:** Baseline characteristics of study population Values are expressed as mean ± SD for continuous variables and as frequency (percentage) for categorical variables. Group comparisons were performed using the independent samples t-test for continuous variables and the chi-square test for categorical variables.

Parameter	Patients With CKD (n = 80)	Controls (n = 80)	Test Statistic	P-value
Age (years, mean ± SD)	52.6 ± 11.4	50.8 ± 10.9	t = 1.02	0.312
Male sex, n (%)	45 (56.3)	43 (53.8)	χ² = 0.10	0.842
Hypertension, n (%)	50 (62.5)	12 (15.0)	χ² = 38.03	<0.001
Diabetes mellitus, n (%)	39 (48.8)	10 (12.5)	χ² = 24.74	<0.001

Renal function parameters

Renal function markers were significantly altered in CKD patients. Mean serum creatinine levels were markedly elevated in CKD patients (4.6 ± 1.8 mg/dL) compared to controls (0.9 ± 0.2 mg/dL; p < 0.001). Similarly, eGFR was significantly reduced in CKD patients (28.4 ± 10.2 mL/min/1.73 m²) compared to controls (96.2 ± 12.5 mL/min/1.73 m²; p < 0.001) (Table 2).

**Table 2:** Renal function parameters in CKD patients and healthy controls Data are expressed as mean ± SD. Comparisons between CKD patients and healthy controls were carried out using the independent samples t-test. CKD: Chronic kidney disease; eGFR: Estimated glomerular filtration rate.

Parameter	Reference Range	Patients With CKD (n = 80)	Controls (n = 80)	Test Statistic	P-value
Serum creatinine (mg/dL)	0.6-1.2 mg/dL	4.6 ± 1.8	0.9 ± 0.2	t = 18.25	<0.001
Blood urea (mg/dL)	15-40 mg/dL	86.3 ± 24.5	28.7 ± 9.4	t = 21.84	<0.001
eGFR (mL/min/1.73 m²)	≥90 mL/min/1.73 m²	28.4 ± 10.2	96.2 ± 12.5	t = -37.43	<0.001

Oxidative stress markers

Markers of oxidative stress were significantly elevated in CKD patients. Serum MDA levels were nearly doubled in CKD patients (5.8 ± 1.2 nmol/mL) compared to controls (2.9 ± 0.8 nmol/mL; p < 0.001). In contrast, antioxidant enzyme levels were markedly reduced (Table 3).

**Table 3:** Oxidative stress markers in CKD patients and healthy controls Data are presented as mean ± SD. Comparisons between the two groups were performed using the independent samples t-test. CKD: Chronic kidney disease; MDA: Malondialdehyde; SOD: Superoxide dismutase.

Parameter	Patients With CKD (n = 80)	Controls (n = 80)	Test Statistic	P-value
MDA (nmol/mL)	5.8 ± 1.2	2.9 ± 0.8	t = 17.99	<0.001
SOD (U/mL)	1.9 ± 0.6	3.8 ± 0.9	t = -15.71	<0.001
Catalase (kU/L)	28.5 ± 6.7	46.2 ± 8.1	t = -15.06	<0.001

SOD activity was significantly lower in CKD patients (1.9 ± 0.6 U/mL) than that in controls (3.8 ± 0.9 U/mL; p < 0.001). Catalase activity also showed a significant decline in CKD patients (28.5 ± 6.7 kU/L vs 46.2 ± 8.1 kU/L; p < 0.001).

Nitrosative stress marker

Serum nitric oxide levels were markedly elevated in patients with CKD when compared with the healthy control group. The mean nitric oxide concentration in the CKD group was 62.4 ± 10.5 µmol/L, whereas the corresponding value in controls was 34.7 ± 7.9 µmol/L. This difference was statistically highly significant (p < 0.001), suggesting that patients with CKD were exposed to greater nitrosative stress (Table 4).

**Table 4:** Nitrosative stress marker in CKD patients and healthy controls Data are presented as mean ± SD. Comparison between the two groups was performed using the independent samples t-test. CKD: Chronic kidney disease.

Parameter	Patients With CKD (n = 80)	Controls (n = 80)	Test Statistic	P-value
Nitric oxide (µmol/L)	62.4 ± 10.5	34.7 ± 7.9	t = 18.86	<0.001

These findings support the view that altered nitric oxide metabolism may be involved in the pathophysiology of renal dysfunction and may contribute to ongoing cellular and vascular injury in CKD.

Correlation analysis

The present study showed a clear association between renal function and the biochemical markers of oxidative stress. Estimated glomerular filtration rate (eGFR), which reflects the level of kidney function, demonstrated a significant negative correlation with malondialdehyde (MDA) levels (r = −0.62, p < 0.001) (Table 5).

**Table 5:** Pearson’s correlation coefficients showing the association of oxidative and nitrosative stress markers with renal function parameters among CKD patients CKD: Chronic kidney disease; MDA: Malondialdehyde; SOD: Superoxide dismutase.

Parameter	Correlation Coefficient (r)	P-value
MDA	−0.62	<0.001
SOD	+0.58	<0.001
Catalase	+0.54	<0.001
Nitric oxide	−0.49	<0.001

**Figure 1:**
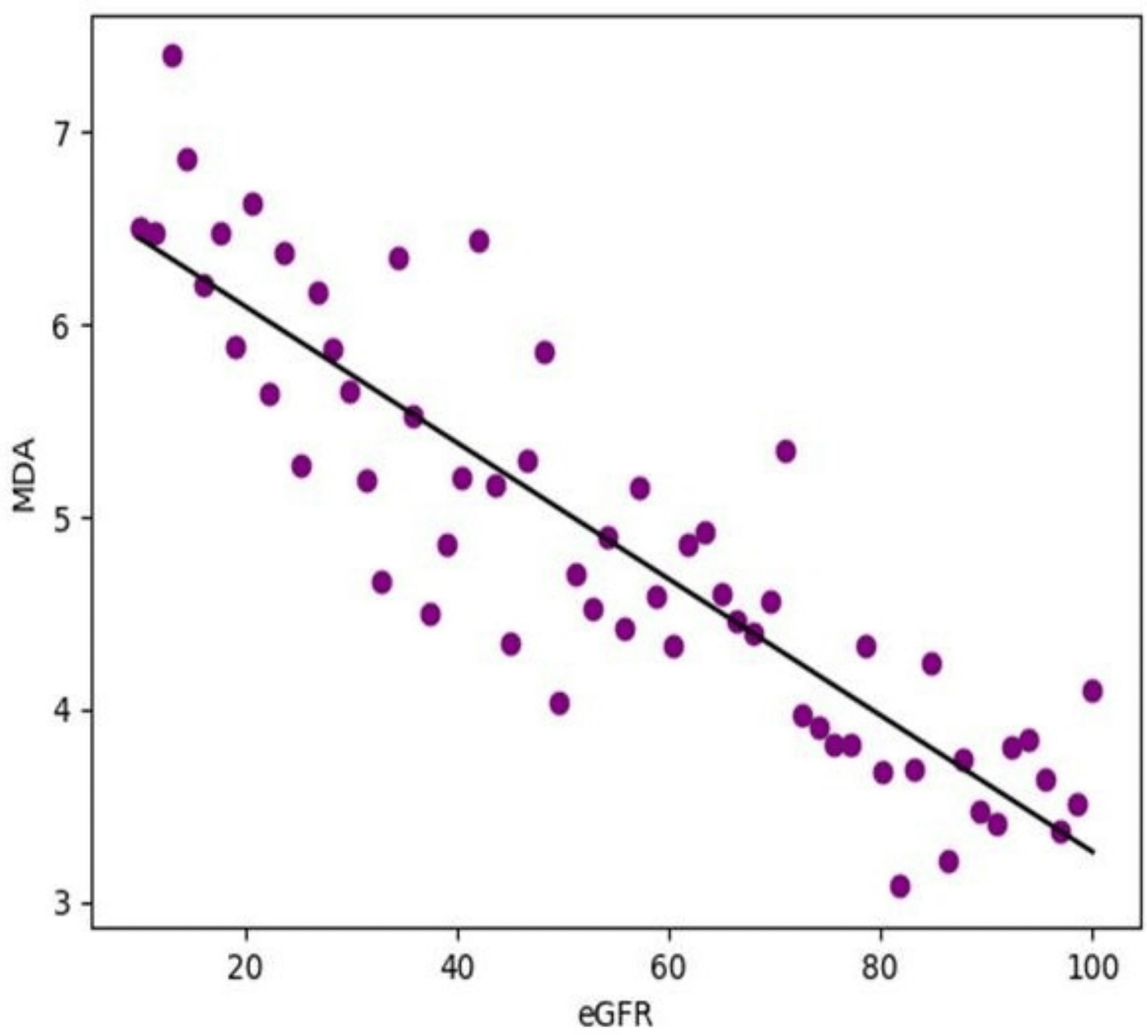
Correlation between eGFR (mL/min/1.73 m²) and MDA (nmol/mL) among CKD patients Pearson’s correlation analysis showed a significant inverse relationship between eGFR and MDA levels (r = -0.62, p < 0.001). CKD: Chronic kidney disease; eGFR: Estimated glomerular filtration rate; MDA: Malondialdehyde.

This indicates that as kidney function declines, MDA levels tend to rise. Since MDA is a widely used marker of lipid peroxidation, this finding suggests that worsening renal impairment is accompanied by increasing oxidative damage to cellular lipids. In other words, patients with lower eGFR values were more likely to have higher oxidative stress burden (Figure 1).

**Figure 2:**
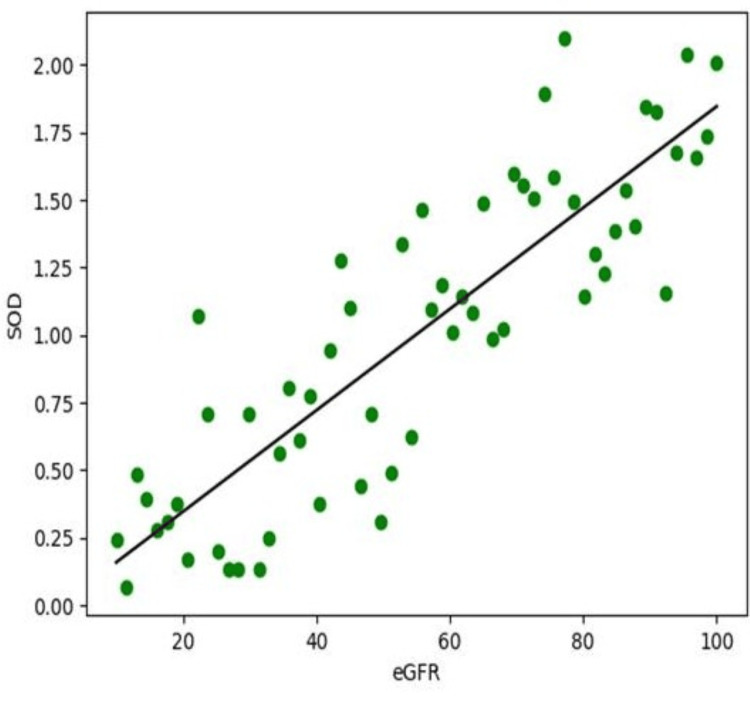
Correlation between eGFR (mL/min/1.73 m²) and SOD (U/mL) among CKD patients Pearson’s correlation analysis showed a significant positive relationship between eGFR and SOD levels (r = 0.58, p < 0.001). CKD: Chronic kidney disease; eGFR: Estimated glomerular filtration rate; SOD: Superoxide dismutase.

On the other hand, the antioxidant enzymes, superoxide dismutase (SOD) and catalase, showed significant positive correlations with eGFR. SOD demonstrated a correlation coefficient of r = 0.58 (p < 0.001), while catalase showed a correlation coefficient of r = 0.54 (p < 0.001). These positive relationships indicate that better preserved renal function is associated with higher antioxidant enzyme activity. As eGFR decreases, the levels of these protective enzymes also tend to decline, reflecting a progressive weakening of the body’s natural antioxidant defence system in CKD (Figure 2).

Taken together, these findings highlight an important pattern in CKD. With deterioration in renal function, oxidative injury becomes more pronounced, while antioxidant protection becomes progressively compromised. The inverse relationship between eGFR and MDA, along with the direct relationship of eGFR with SOD and catalase, supports the concept that oxidative stress is closely linked to the severity of CKD. These correlations also suggest that oxidative stress markers may provide useful additional information regarding disease progression and the underlying biochemical disturbances seen in patients with impaired kidney function, as presented in Table 5.

## Discussion

The present study evaluated oxidative and nitrosative stress status in patients with CKD and compared the findings with those of healthy controls. The results showed a clear shift towards a pro-oxidant state in CKD, as reflected by increased lipid peroxidation, raised nitric oxide levels, and reduced antioxidant enzyme activity. These findings support the view that redox imbalance is closely involved in the biological changes associated with CKD and may contribute to progressive renal dysfunction.

A major finding of this study was the significant increase in serum malondialdehyde levels among CKD patients. MDA is a commonly used marker of lipid peroxidation and indicates oxidative injury to cellular membranes. The higher MDA levels observed in the CKD group suggest enhanced free radical-mediated damage. This finding is consistent with earlier studies reporting increased oxidative damage in CKD due to retention of uremic toxins, mitochondrial dysfunction, chronic inflammation, and impaired antioxidant defence [1,2]. As kidney function declines, the ability to eliminate pro-oxidant molecules decreases, thereby intensifying oxidative stress and cellular injury.

The antioxidant defence system was also found to be significantly impaired in CKD patients. In the present study, superoxide dismutase and catalase activities were lower in CKD patients compared with controls. SOD is responsible for converting superoxide radicals into hydrogen peroxide, while catalase further breaks down hydrogen peroxide into water and oxygen. Reduced activity of these enzymes indicates that the endogenous antioxidant system is under strain and may be insufficient to neutralise the excess free radicals generated in CKD. Similar reductions in antioxidant enzyme activity have been reported in previous studies, in which lower SOD and catalase levels were associated with worsening renal function and increased oxidative injury [3,4].

Another important observation was the increase in nitric oxide levels among CKD patients, suggesting the presence of nitrosative stress. Nitric oxide has an important physiological role in maintaining vascular tone, renal blood flow, and endothelial function. However, under conditions of oxidative stress, nitric oxide can react with superoxide radicals to form peroxynitrite, a highly reactive compound that damages proteins, lipids, DNA, and endothelial cells. This mechanism may contribute to endothelial dysfunction, vascular injury, and inflammation in CKD [5,6]. The elevated nitric oxide levels in this study are in agreement with previous reports describing altered nitric oxide metabolism and endothelial damage in renal disease [7].

The correlation analysis adds clinical relevance to the biochemical findings. A significant negative correlation between eGFR and MDA indicates that lipid peroxidation increases as renal function declines. In contrast, the positive correlation between eGFR and antioxidant enzymes such as SOD and catalase suggests that antioxidant capacity decreases with increasing severity of kidney dysfunction. These findings are consistent with earlier studies showing that oxidative stress is closely linked with CKD severity and progression [8,9]. The observed relationship between redox markers and eGFR also supports the usefulness of these biomarkers as additional indicators of disease burden, although they cannot replace standard renal function tests.

The interaction between oxidative stress and inflammation is also important in CKD. CKD is often associated with persistent low-grade inflammation, which promotes the generation of ROS and RNS. Inflammatory mediators can activate enzymes such as NADPH oxidase, leading to sustained oxidative injury [2,10]. At the same time, oxidative stress can further stimulate inflammatory pathways, creating a self-perpetuating cycle. This cycle may promote endothelial dysfunction, renal fibrosis, tubular injury, and progressive nephron loss.

The present study has several strengths. The case-control design was appropriate for comparing oxidative and nitrosative stress markers between CKD patients and healthy controls. The inclusion and exclusion criteria were clearly defined, which helped reduce selection-related variability. Multiple markers were assessed, including MDA, nitric oxide, SOD, and catalase, allowing a broader evaluation of both oxidant burden and antioxidant defence. In addition, correlation analysis with eGFR improved the clinical relevance of the findings by linking biochemical changes with the severity of renal impairment.

At the same time, the findings should be interpreted with caution. Oxidative and nitrosative stress markers can be influenced by several factors other than CKD itself. Dietary antioxidant intake, smoking, alcohol use, physical activity, obesity, associated comorbidities, and medication use may affect the measured biomarker levels. Although major confounding illnesses were excluded, these lifestyle and treatment-related variables were not assessed in detail. Therefore, their possible influence on the results cannot be fully ruled out. Future studies should include a more detailed assessment of dietary habits, lifestyle factors, medication exposure, and inflammatory status to better clarify the independent relationship between CKD and redox imbalance.

From a clinical perspective, the findings suggest that oxidative and nitrosative stress markers may provide additional information regarding disease severity in CKD. Biomarkers such as MDA, nitric oxide, SOD, and catalase may reflect the biochemical stress burden associated with renal dysfunction. However, their routine clinical use requires further validation through larger, multicentre, and longitudinal studies. Although antioxidant-based therapies have been explored in CKD, the clinical benefit has been variable, and more targeted approaches may be required to modify redox pathways effectively [4,11].

The broader significance of these findings lies in the link between oxidative stress, renal dysfunction, and cardiovascular complications. CKD patients are at increased risk of endothelial dysfunction, vascular stiffness, atherosclerosis, and cardiovascular mortality. Oxidative and nitrosative stress may contribute to these complications by promoting vascular inflammation, impaired nitric oxide signalling, and endothelial injury [6,12]. Therefore, a better understanding of redox imbalance in CKD may help in identifying patients at higher risk and in developing supportive strategies to reduce disease-related complications.

Overall, the findings of this study are in line with previous literature showing increased oxidative injury, altered nitric oxide metabolism, and weakened antioxidant defence in CKD [3,8]. While the study supports an association between redox imbalance and renal dysfunction, the cross-sectional design prevents any conclusion regarding causality. Further prospective studies are needed to determine whether these biomarkers can predict CKD progression or response to therapeutic interventions.

Limitations of the study

This study has certain limitations. First, the cross-sectional design limits the ability to establish a causal relationship between oxidative stress, nitrosative stress, and CKD progression. The study demonstrates an association between redox imbalance and reduced renal function, but it cannot determine whether these biochemical changes are a cause or consequence of worsening kidney disease.

Second, the study was conducted at a single centre with a modest sample size. Although the findings were statistically significant, the results may not fully represent CKD patients from different geographic, ethnic, dietary, or clinical backgrounds. Larger multicentre studies would improve the generalisability of the observations.

Third, possible confounding variables were not assessed in detail. Factors such as dietary antioxidant intake, smoking, alcohol consumption, physical activity, body mass index, medication use, glycaemic control, blood pressure control, and associated comorbidities may influence oxidative and nitrosative stress markers. Lack of detailed adjustment for these variables may have affected the measured biomarker levels.

Fourth, only a selected panel of biomarkers was included. The study assessed MDA, nitric oxide, SOD, and catalase, but additional markers such as reduced glutathione, glutathione peroxidase, advanced oxidation protein products, protein carbonyls, myeloperoxidase, interleukins, tumour necrosis factor-alpha, and high-sensitivity C-reactive protein were not measured. Inclusion of these markers could have provided a more comprehensive understanding of oxidative stress, nitrosative stress, and inflammation in CKD.

Fifth, the study did not include longitudinal follow-up. Serial measurement of oxidative and nitrosative stress markers would have helped determine whether these biomarkers change with CKD progression, treatment, dialysis status, or improvement in renal function parameters.

Finally, although standard biochemical methods were used, pre-analytical and biological variability cannot be completely excluded. Differences in sample collection time, dietary status, storage duration, and individual metabolic variation may have influenced biomarker concentrations. Despite these limitations, the study provides useful evidence supporting the association of oxidative and nitrosative stress with renal dysfunction in CKD.

## Conclusions

The present study shows that patients with CKD had a measurable redox imbalance when compared with healthy controls. Higher levels of MDA and NO, together with lower superoxide dismutase and catalase activity, indicate increased oxidative and nitrosative stress in CKD. The observed correlations with eGFR suggest that these biomarker changes are associated with the degree of renal dysfunction. However, because of the cross-sectional design, these findings should be interpreted as associations and not as evidence of causality or disease progression. Assessment of these markers may provide additional biochemical insight into CKD status, while larger longitudinal studies are needed to clarify their prognostic value and possible therapeutic relevance.
